# 3-Methoxy-4-(4-nitro­benz­yloxy)­benzaldehyde

**DOI:** 10.1107/S1600536808036015

**Published:** 2008-11-08

**Authors:** Mei Li, Xin Chen

**Affiliations:** a College of Sciences, Tianjin University of Science and Technology, Tianjin 300457, People’s Republic of China

## Abstract

In the title compound, C_15_H_13_NO_5_, the vanillin group makes a dihedral angle of 4.95 (8)° with the benzene ring of the nitro­benzene group. The packing is stabilized by weak, non-classical inter­molecular C—H⋯O inter­actions which link mol­ecules into chains running along the *c* axis.

## Related literature

For general background on Schiff bases, see: Kahwa *et al.* (1986 ▶); Santos *et al.* (2001 ▶). For bond-length data, see: Allen *et al.* (1987 ▶);
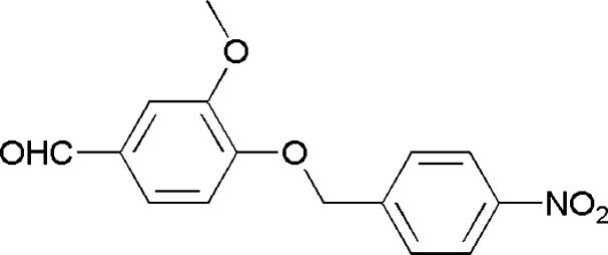


## Experimental

### 

#### Crystal data

C_15_H_13_NO_5_
*M* *_r_* = 287.26
Orthorhombic, 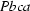

*a* = 13.743 (3) Å
*b* = 12.526 (3) Å
*c* = 16.384 (3) Å
*V* = 2820.4 (10) Å^3^
*Z* = 8
Mo *K*α radiation
μ = 0.10 mm^−1^
*T* = 294 (2) K
0.23 × 0.18 × 0.12 mm

#### Data collection

Bruker SMART APEX CCD area-detector diffractometer
Absorption correction: multi-scan (*SADABS*; Sheldrick, 1996 ▶) *T*_min_ = 0.932, *T*_max_ = 0.988
15172 measured reflections
2877 independent reflections
1540 reflections with *I* > 2σ(*I*)
*R*_int_ = 0.045

#### Refinement

*R*[*F* ^2^ > 2σ(*F* ^2^)] = 0.044
*wR*(*F* ^2^) = 0.132
*S* = 0.99
2877 reflections
192 parameters
H-atom parameters constrained
Δρ_max_ = 0.16 e Å^−3^
Δρ_min_ = −0.17 e Å^−3^

### 

Data collection: *SMART* (Bruker, 1999 ▶); cell refinement: *SAINT* (Bruker, 1999 ▶); data reduction: *SAINT*; program(s) used to solve structure: *SHELXS97* (Sheldrick, 2008 ▶); program(s) used to refine structure: *SHELXL97* (Sheldrick, 2008 ▶); molecular graphics: *SHELXTL* (Sheldrick, 2008 ▶); software used to prepare material for publication: *SHELXTL*.

## Supplementary Material

Crystal structure: contains datablocks I, 71007a. DOI: 10.1107/S1600536808036015/at2666sup1.cif

Structure factors: contains datablocks I. DOI: 10.1107/S1600536808036015/at2666Isup2.hkl

Additional supplementary materials:  crystallographic information; 3D view; checkCIF report

## Figures and Tables

**Table 1:** Hydrogen-bond geometry (Å, °)

*D*—H⋯*A*	*D*—H	H⋯*A*	*D*⋯*A*	*D*—H⋯*A*
C14—H14⋯O5^i^	0.93	2.60	3.405 (3)	146

Symmetry code: (i) 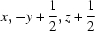.

**Fig. 1.**
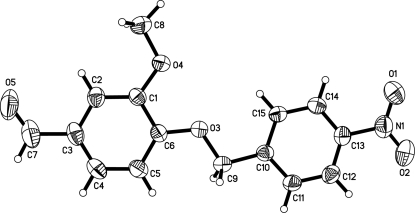
The structure of (I), with displacement ellipsoids for non-H atoms drawn at the 30% probability level.

**Fig. 2.**
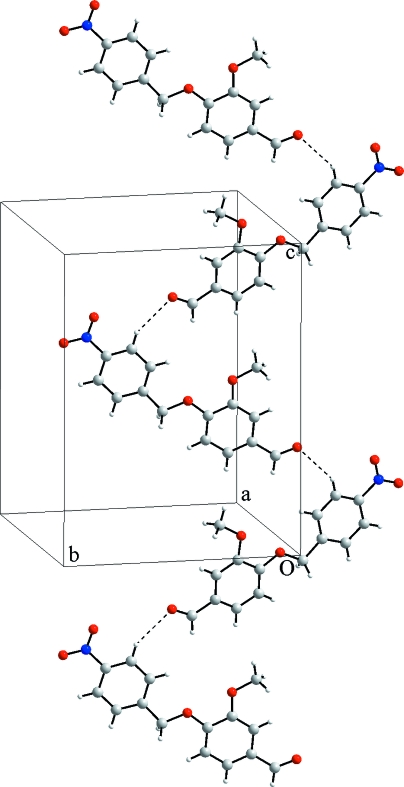
Packing diagram for (I), with H bonds drawn as dashed lines.

## supplementary crystallographic information

### Comment

Schiff-base ligands have received a good deal of attention in biology and chemistry (Kahwa *et al.*, 1986). Many Schiff base derivatives have been synthesized and employed to develop protein and enzyme mimics (Santos *et al.*, 2001). As a part of our interest in the coordination properties of Schiff bases functioning as ligands, we investigated the title compound, (I), used as a precursor in the preparation of Schiff bases.

In the title molecule (Fig. 1), bond lengths and angles are within normal ranges (Allen *et al.*, 1987). The vanillin group (C1—C7/O3/O4) is essentially planar (except the methyl H atoms), with an r.m.s. deviation for fitted atoms of 0.035 (3) Å. This group makes a dihedral angle of 4.95 (8)° with the benzene ring (C10—C15) of the nitrobenzene group.

The crystal packing is stabilized by weak, non-classical intermolecular C14—H14···O5═C7 interactions that link adjacent molecules into one-dimensional chains running along the *c* axis (Table 1, Fig. 2).

### Experimental

An anhydrous acetonitrile solution (100 ml) of 4-hydroxy-3-methoxybenzaldehyde (1.52 g, 10 mmol) was added dropwise to a solution (50 ml) of 1-(bromomethyl)-4-nitrobenzene (2.16 g, 10 mmol) and pyridine (0.79 g, 10 mmol) in acetonitrile, in 30 min., and the mixture refluxed for 24 h under nitrogen atmosphere. The solvent was removed and the resultant mixture poured into ice-water (100 ml). The yellow precipitate was then isolated and recrystallized from acetonitrile, and then dried in a vacuum to give the pure compound in 74% yield. Pale-yellow single crystals of (I) suitable for X-ray analysis were obtained by slow evaporation of an acetonitrile solution.

### Refinement

The H atoms were included in calculated positions and refined using a riding model approximation. Constrained C—H bond lengths and isotropic U parameters: 0.93 Å and *U*_iso_(H) = 1.2*U*_eq_(C) for C*sp*^2^—H; 0.97 Å and *U*_iso_(H) = 1.2*U*_eq_(C) for methylene C—H; 0.96 Å and *U*_iso_(H) = 1.5*U*_eq_(C) for methyl C—H.

### Crystal data

**Table:**

C_15_H_13_NO_5_	*F*_000_ = 1200
*M**_r_* = 287.26	*D*_x_ = 1.353 Mg m^−^^3^
Orthorhombic, *P**b**c**a*	Mo *K*α radiation λ = 0.71073 Å
Hall symbol: -P 2ac 2ab	Cell parameters from 3156 reflections
*a* = 13.743 (3) Å	θ = 2.2–26.5º
*b* = 12.526 (3) Å	µ = 0.10 mm^−^^1^
*c* = 16.384 (3) Å	*T* = 294 (2) K
*V* = 2820.4 (10) Å^3^	Block, pale-yellow
*Z* = 8	0.23 × 0.18 × 0.12 mm

### Data collection

**Table:**

Bruker SMART APEX CCD area-detector diffractometer	2877 independent reflections
Radiation source: fine-focus sealed tube	1540 reflections with *I* > 2σ(*I*)
Monochromator: graphite	*R*_int_ = 0.045
*T* = 294(2) K	θ_max_ = 26.4º
φ and ω scans	θ_min_ = 2.5º
Absorption correction: multi-scan(SADABS; Sheldrick, 1996)	*h* = −16→17
*T*_min_ = 0.932, *T*_max_ = 0.988	*k* = −14→15
15172 measured reflections	*l* = −20→18

### Refinement

**Table:**

Refinement on *F*^2^	Hydrogen site location: inferred from neighbouring sites
Least-squares matrix: full	H-atom parameters constrained
*R*[*F*^2^ > 2σ(*F*^2^)] = 0.044	*w* = 1/[σ^2^(*F*_o_^2^) + (0.0424*P*)^2^ + 1.1393*P*] where *P* = (*F*_o_^2^ + 2*F*_c_^2^)/3
*wR*(*F*^2^) = 0.132	(Δ/σ)_max_ = 0.001
*S* = 0.99	Δρ_max_ = 0.16 e Å^−^^3^
2877 reflections	Δρ_min_ = −0.17 e Å^−^^3^
192 parameters	Extinction correction: SHELXL, Fc^*^=kFc[1+0.001xFc^2^λ^3^/sin(2θ)]^-1/4^
Primary atom site location: structure-invariant direct methods	Extinction coefficient: 0.0017 (5)
Secondary atom site location: difference Fourier map	

### Special details

**Table:**

Geometry. All e.s.d.'s (except the e.s.d. in the dihedral angle between two l.s. planes) are estimated using the full covariance matrix. The cell e.s.d.'s are taken into account individually in the estimation of e.s.d.'s in distances, angles and torsion angles; correlations between e.s.d.'s in cell parameters are only used when they are defined by crystal symmetry. An approximate (isotropic) treatment of cell e.s.d.'s is used for estimating e.s.d.'s involving l.s. planes.
Refinement. Refinement of *F*^2^ against ALL reflections. The weighted *R*-factor *wR* and goodness of fit *S* are based on *F*^2^, conventional *R*-factors *R* are based on *F*, with *F* set to zero for negative *F*^2^. The threshold expression of *F*^2^ > 2σ(*F*^2^) is used only for calculating *R*-factors(gt) *etc*. and is not relevant to the choice of reflections for refinement. *R*-factors based on *F*^2^ are statistically about twice as large as those based on *F*, and *R*- factors based on ALL data will be even larger.

### Fractional atomic coordinates and isotropic or equivalent isotropic displacement parameters (Å^2^)

**Table:**

	*x*	*y*	*z*	*U*_iso_*/*U*_eq_	
N1	0.14273 (18)	−0.3678 (2)	1.20564 (17)	0.0880 (7)	
O1	0.1166 (2)	−0.34357 (19)	1.27401 (14)	0.1320 (9)	
O2	0.1674 (2)	−0.45767 (19)	1.18670 (15)	0.1303 (9)	
O3	0.11868 (11)	0.05467 (12)	0.99132 (8)	0.0671 (5)	
O4	0.07376 (11)	0.23118 (12)	1.05892 (9)	0.0705 (5)	
O5	0.1082 (2)	0.5139 (2)	0.82786 (17)	0.1500 (12)	
C1	0.09685 (14)	0.23830 (18)	0.97787 (13)	0.0570 (5)	
C2	0.09799 (15)	0.33013 (19)	0.93261 (15)	0.0671 (6)	
H2	0.0826	0.3950	0.9570	0.081*	
C3	0.12223 (16)	0.3267 (2)	0.84953 (16)	0.0739 (7)	
C4	0.14582 (18)	0.2313 (2)	0.81422 (15)	0.0772 (7)	
H4	0.1626	0.2294	0.7592	0.093*	
C5	0.14517 (17)	0.1375 (2)	0.85877 (14)	0.0704 (7)	
H5	0.1610	0.0730	0.8340	0.085*	
C6	0.12076 (15)	0.14069 (17)	0.94062 (13)	0.0572 (6)	
C7	0.1230 (2)	0.4259 (3)	0.8018 (2)	0.1087 (12)	
H7	0.1361	0.4199	0.7463	0.130*	
C8	0.0516 (2)	0.3282 (2)	1.10024 (16)	0.0976 (10)	
H8A	0.1071	0.3747	1.0985	0.146*	
H8B	0.0354	0.3130	1.1560	0.146*	
H8C	−0.0027	0.3623	1.0741	0.146*	
C9	0.14047 (18)	−0.04690 (17)	0.95782 (13)	0.0680 (6)	
H9A	0.2033	−0.0447	0.9309	0.082*	
H9B	0.0917	−0.0660	0.9176	0.082*	
C10	0.14211 (15)	−0.12841 (17)	1.02476 (13)	0.0562 (5)	
C11	0.16208 (16)	−0.23375 (19)	1.00431 (14)	0.0665 (6)	
H11	0.1746	−0.2516	0.9502	0.080*	
C12	0.16355 (17)	−0.31198 (19)	1.06322 (16)	0.0707 (7)	
H12	0.1777	−0.3824	1.0496	0.085*	
C13	0.14380 (16)	−0.28411 (19)	1.14254 (15)	0.0637 (6)	
C14	0.12433 (18)	−0.18140 (19)	1.16476 (14)	0.0709 (7)	
H14	0.1117	−0.1644	1.2190	0.085*	
C15	0.12358 (17)	−0.10322 (19)	1.10550 (14)	0.0668 (6)	
H15	0.1105	−0.0329	1.1200	0.080*	

### Atomic displacement parameters (Å^2^)

**Table:**

	*U*^11^	*U*^22^	*U*^33^	*U*^12^	*U*^13^	*U*^23^
N1	0.0997 (17)	0.0762 (17)	0.0882 (18)	−0.0093 (13)	−0.0130 (14)	0.0156 (14)
O1	0.212 (3)	0.1117 (17)	0.0727 (14)	−0.0042 (16)	0.0010 (16)	0.0221 (13)
O2	0.177 (2)	0.0729 (15)	0.141 (2)	0.0080 (15)	0.0028 (17)	0.0271 (14)
O3	0.0910 (12)	0.0563 (10)	0.0541 (9)	0.0010 (8)	0.0058 (8)	−0.0018 (7)
O4	0.0939 (12)	0.0657 (10)	0.0520 (9)	0.0107 (8)	0.0013 (8)	−0.0026 (8)
O5	0.175 (3)	0.1065 (19)	0.169 (2)	0.0499 (18)	0.0663 (19)	0.0703 (18)
C1	0.0525 (12)	0.0661 (15)	0.0524 (12)	−0.0001 (10)	−0.0015 (10)	0.0039 (11)
C2	0.0595 (14)	0.0662 (15)	0.0757 (16)	0.0055 (11)	0.0020 (12)	0.0117 (12)
C3	0.0561 (14)	0.0888 (19)	0.0768 (17)	0.0040 (13)	0.0062 (12)	0.0297 (15)
C4	0.0726 (16)	0.104 (2)	0.0555 (14)	−0.0001 (15)	0.0069 (12)	0.0140 (15)
C5	0.0762 (16)	0.0778 (17)	0.0573 (14)	−0.0025 (13)	0.0029 (12)	−0.0004 (13)
C6	0.0584 (13)	0.0597 (14)	0.0536 (13)	−0.0043 (10)	−0.0026 (10)	0.0043 (11)
C7	0.090 (2)	0.122 (3)	0.114 (2)	0.029 (2)	0.0266 (18)	0.057 (2)
C8	0.148 (3)	0.0751 (19)	0.0700 (16)	0.0201 (18)	−0.0029 (17)	−0.0147 (14)
C9	0.0865 (17)	0.0611 (15)	0.0563 (13)	0.0032 (12)	0.0080 (12)	−0.0068 (11)
C10	0.0538 (12)	0.0587 (14)	0.0562 (13)	−0.0018 (10)	0.0038 (10)	−0.0046 (10)
C11	0.0712 (15)	0.0645 (16)	0.0640 (14)	0.0029 (12)	0.0158 (11)	−0.0088 (12)
C12	0.0698 (15)	0.0577 (15)	0.0847 (18)	0.0049 (11)	0.0114 (13)	−0.0043 (13)
C13	0.0598 (13)	0.0628 (15)	0.0685 (15)	−0.0028 (11)	−0.0047 (12)	0.0070 (12)
C14	0.0905 (18)	0.0715 (17)	0.0509 (13)	−0.0038 (13)	−0.0053 (12)	−0.0051 (12)
C15	0.0865 (17)	0.0584 (14)	0.0555 (14)	−0.0008 (12)	−0.0029 (12)	−0.0085 (11)

### Geometric parameters (Å, °)

**Table:**

N1—O1	1.215 (3)	C7—H7	0.9300
N1—O2	1.216 (3)	C8—H8A	0.9600
N1—C13	1.472 (3)	C8—H8B	0.9600
O3—C6	1.361 (2)	C8—H8C	0.9600
O3—C9	1.418 (2)	C9—C10	1.499 (3)
O4—C1	1.368 (2)	C9—H9A	0.9700
O4—C8	1.424 (3)	C9—H9B	0.9700
O5—C7	1.199 (4)	C10—C15	1.384 (3)
C1—C2	1.369 (3)	C10—C11	1.389 (3)
C1—C6	1.406 (3)	C11—C12	1.376 (3)
C2—C3	1.402 (3)	C11—H11	0.9300
C2—H2	0.9300	C12—C13	1.373 (3)
C3—C4	1.367 (3)	C12—H12	0.9300
C3—C7	1.468 (4)	C13—C14	1.364 (3)
C4—C5	1.383 (3)	C14—C15	1.379 (3)
C4—H4	0.9300	C14—H14	0.9300
C5—C6	1.383 (3)	C15—H15	0.9300
C5—H5	0.9300		
			
O1—N1—O2	123.3 (3)	H8A—C8—H8B	109.5
O1—N1—C13	118.2 (3)	O4—C8—H8C	109.5
O2—N1—C13	118.5 (3)	H8A—C8—H8C	109.5
C6—O3—C9	118.02 (16)	H8B—C8—H8C	109.5
C1—O4—C8	117.09 (18)	O3—C9—C10	109.34 (17)
O4—C1—C2	125.7 (2)	O3—C9—H9A	109.8
O4—C1—C6	114.77 (19)	C10—C9—H9A	109.8
C2—C1—C6	119.5 (2)	O3—C9—H9B	109.8
C1—C2—C3	120.2 (2)	C10—C9—H9B	109.8
C1—C2—H2	119.9	H9A—C9—H9B	108.3
C3—C2—H2	119.9	C15—C10—C11	118.9 (2)
C4—C3—C2	119.6 (2)	C15—C10—C9	122.8 (2)
C4—C3—C7	120.9 (3)	C11—C10—C9	118.28 (19)
C2—C3—C7	119.5 (3)	C12—C11—C10	120.7 (2)
C3—C4—C5	121.2 (2)	C12—C11—H11	119.6
C3—C4—H4	119.4	C10—C11—H11	119.6
C5—C4—H4	119.4	C13—C12—C11	118.7 (2)
C4—C5—C6	119.3 (2)	C13—C12—H12	120.7
C4—C5—H5	120.4	C11—C12—H12	120.7
C6—C5—H5	120.4	C14—C13—C12	122.1 (2)
O3—C6—C5	125.1 (2)	C14—C13—N1	118.8 (2)
O3—C6—C1	114.76 (19)	C12—C13—N1	119.1 (2)
C5—C6—C1	120.2 (2)	C13—C14—C15	118.9 (2)
O5—C7—C3	126.0 (3)	C13—C14—H14	120.5
O5—C7—H7	117.0	C15—C14—H14	120.5
C3—C7—H7	117.0	C14—C15—C10	120.7 (2)
O4—C8—H8A	109.5	C14—C15—H15	119.7
O4—C8—H8B	109.5	C10—C15—H15	119.7
			
C8—O4—C1—C2	−1.8 (3)	C2—C3—C7—O5	3.3 (5)
C8—O4—C1—C6	178.3 (2)	C6—O3—C9—C10	175.41 (18)
O4—C1—C2—C3	−179.5 (2)	O3—C9—C10—C15	0.6 (3)
C6—C1—C2—C3	0.4 (3)	O3—C9—C10—C11	179.86 (19)
C1—C2—C3—C4	−0.8 (3)	C15—C10—C11—C12	−0.1 (3)
C1—C2—C3—C7	179.9 (2)	C9—C10—C11—C12	−179.4 (2)
C2—C3—C4—C5	0.7 (4)	C10—C11—C12—C13	0.7 (3)
C7—C3—C4—C5	−179.9 (2)	C11—C12—C13—C14	−1.0 (4)
C3—C4—C5—C6	−0.4 (4)	C11—C12—C13—N1	178.4 (2)
C9—O3—C6—C5	−1.8 (3)	O1—N1—C13—C14	6.0 (4)
C9—O3—C6—C1	179.01 (19)	O2—N1—C13—C14	−174.4 (2)
C4—C5—C6—O3	−179.1 (2)	O1—N1—C13—C12	−173.5 (3)
C4—C5—C6—C1	0.0 (3)	O2—N1—C13—C12	6.1 (4)
O4—C1—C6—O3	−1.0 (3)	C12—C13—C14—C15	0.6 (4)
C2—C1—C6—O3	179.12 (19)	N1—C13—C14—C15	−178.8 (2)
O4—C1—C6—C5	179.83 (19)	C13—C14—C15—C10	0.1 (4)
C2—C1—C6—C5	−0.1 (3)	C11—C10—C15—C14	−0.4 (3)
C4—C3—C7—O5	−176.1 (3)	C9—C10—C15—C14	178.9 (2)

### Hydrogen-bond geometry (Å, °)

**Table:**

*D*—H···*A*	*D*—H	H···*A*	*D*···*A*	*D*—H···*A*
C14—H14···O5^i^	0.93	2.60	3.405 (3)	146

Symmetry codes: (i) *x*, −*y*+1/2, *z*+1/2.

## References

[bb1] Allen, F. H., Kennard, O., Watson, D. G., Brammer, L., Orpen, A. G. & Taylor, R. (1987). *J. Chem. Soc. Perkin Trans. 2*, pp. S1–19.

[bb2] Bruker (1999). *SMART* and *SAINT* for Windows NT. Bruker AXS Inc., Madison, Wisconsin, USA.

[bb3] Kahwa, I. A., Selbin, J., Hsieh, T. C.-Y. & Laine, R. A. (1986). *Inorg. Chim. Acta*, **118**, 179–185.

[bb4] Santos, M. L. P., Bagatin, I. A., Pereira, E. M. & Ferreira, A. M. D. C. (2001). *J. Chem. Soc. Dalton Trans.* pp. 838–844.

[bb5] Sheldrick, G. M. (1996). *SADABS* University of Göttingen, Germany.

[bb6] Sheldrick, G. M. (2008). *Acta Cryst.* A**64**, 112–122. 10.1107/S0108767307043930 18156677

